# Insights from single-molecule force spectroscopy into chromatin topology

**DOI:** 10.1007/s12551-026-01427-w

**Published:** 2026-03-14

**Authors:** Luuk J. C. Daris, Jorine M. Eeftens

**Affiliations:** Department of Cell Biology, Radboud Institute for Molecular Life Sciences, Radboud University, Geert Grooteplein-Zuid 26-28, 6525 GA Nijmegen, The Netherlands

**Keywords:** Chromatin, Single-molecule biophysics, DNA organization, Nucleosomes

## Abstract

The organization of DNA into chromatin within the eukaryotic nucleus represents one of the most intricate forms of biological packaging. This organization is inherently hierarchical, spanning multiple length scales: from DNA wrapping around histone octamers to form nucleosomes, to the folding of nucleosome arrays and the establishment of higher-order chromatin domains. Understanding how these scales interconnect is essential for explaining how cells regulate gene expression and replicate their genomes. Single-molecule force spectroscopy has provided powerful mechanistic insights into this architecture by enabling direct measurements of the forces that govern chromatin folding and unfolding. These approaches have clarified how chromatin fibers behave under tension and how nucleosome-level interactions contribute to fiber compaction and stability. In this review, we summarize key discoveries enabled by such techniques and discuss emerging opportunities for probing chromatin in increasingly complex and biologically relevant contexts.

## Introduction

Chromatin is hierarchically organized to achieve a delicate balance: it must be compact enough to fit within the confined volume of the nucleus, yet sufficiently accessible to allow dynamic control of gene activity and genome maintenance (Misteli 2020). Each level of chromatin organization, ranging from nucleosome positioning and fiber folding to higher-order domains and chromosome territories, contributes to the regulation of essential processes such as transcription, DNA replication, and DNA repair. Understanding these structural features remains a central problem in molecular biology. In this context, single-molecule force spectroscopy approaches have been particularly valuable, as they enable direct interrogation of chromatin mechanics, folding, and conformational transitions at nanometer resolution, offering key insights into the physical principles underlying genome regulation.

While its strength lies in probing one molecule at a time, force spectroscopy can nevertheless investigate chromatin organization across multiple levels of structural complexity, from single nucleosomes to nucleosome arrays and gene-scale chromatin fibers. It is therefore a valuable method to converge insights from complementary approaches such as single-molecule FRET (smFRET), chromosome conformation capture (3C) or related sequencing-based methods, and live-cell imaging. While smFRET is suitable to infer local conformational dynamics of nucleosomes on angstrom-scale distances (Koopmans et al. 2007; Kilic et al. 2018; Gansen et al. 2018), it lacks the ability to probe long-range elastic properties or energy landscapes under load. Chromosome conformation capture (3C) or related approaches such as Hi-C are tailored to study genomic connectivity and contact probability in the nucleus (Belton et al. 2012). However, such sequencing-based methods provide a static view of proximity of genomic elements without accounting for physical tension or mechanical stability of interactions. Live-cell imaging methods such as live fluorescence in situ hybridization (live-FISH) (Liu et al. 2025) or super-resolution microscopy techniques like stochastic optical reconstruction microscopy (STORM) (Xu et al. 2018) are appropriate to study mesoscale chromatin organization with high resolution in the crowded nuclear environment, but lack the ability to directly infer the molecular interactions that drive genome organization.

Single-molecule force spectroscopy provides the unique ability to directly quantify the mechanical stability, tension, elastic properties, and dynamics that fundamentally drive and maintain genome organization, thereby complementing approaches that focus on defined scales. This review aims to provide an overview of the findings gained from single-molecule force spectroscopy and provide perspective for further opportunities in studying genome organization.

## From nucleosomes to chromatin fibers

**Fig. 1:**
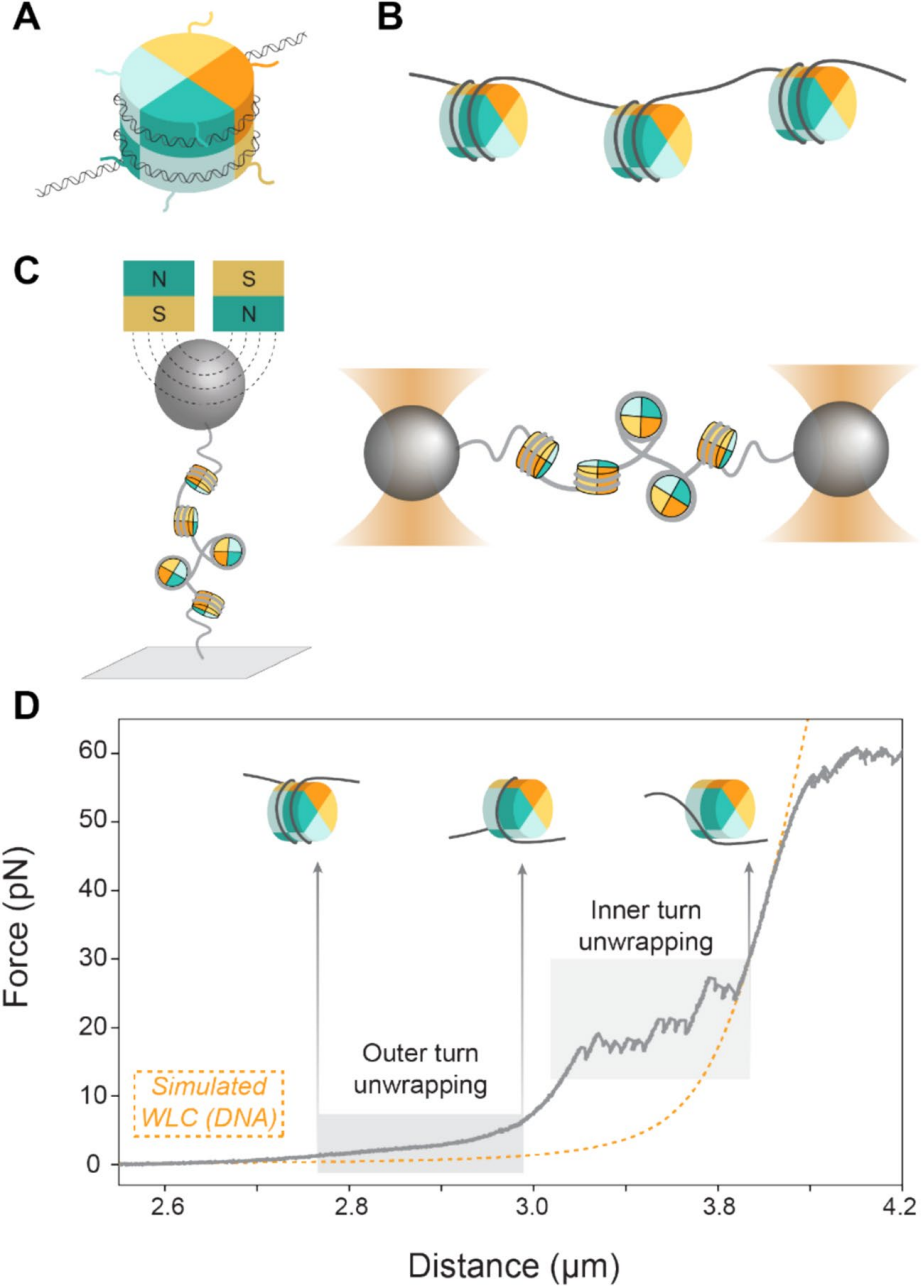
Nucleosomal wrapping quantified by force spectroscopy. **A.** The nucleosome is composed of approximately 147 bp of DNA wrapped around an octamer of histone proteins. **B.** Adjacent nucleosomes are coupled by short stretches of linker DNA to form a nucleosomal array. Tails are omitted for simplicity. **C.** Schematics of the most commonly used force spectroscopy techniques: magnetic tweezers (left), and optical tweezers (right). **D.** Typical force extension curve of an unfolding chromatin array (gray). A simulated worm-like chain (WLC) with a similar contour length as the unfolded chromatin fiber is shown to compare to naked DNA

The most fundamental level of genome organization is the formation of nucleosomes, each composed of approximately 147 bp of DNA wrapped around an octamer of histone proteins **(**Fig. 1A**)**. The histone octamer consists of two copies of each of the core histone proteins H2A, H2B, H3 and H4. Histone proteins have flexible, unstructured tails that protrude from the nucleosome. These tails are the main sites for post-translational modifications (PTMs). DNA wraps around the histone octamer in about 1.7 left-handed superhelical turns (Luger et al. 1997). Adjacent nucleosomes are coupled by short stretches of linker DNA to create a nucleosomal array, forming the structural basis of chromatin fibers (Fletcher and Hansen 1996) **(**Fig. 1B**)**.

Cryo-EM and crystallography have been essential to characterize structural features of chromatin in detail. Still, they provide an inherently static description, while chromatin is a dynamic structure. Biophysical approaches have greatly aided in providing a dynamic description of the chromatin fiber. Specifically, single-molecule force spectroscopy has been a valuable tool to study the mechanical properties of chromatin, as it enables manipulation of individual fibers with high spatial and temporal resolution. The two main methods are magnetic tweezers and optical tweezers **(**Fig. 1C**)**. Both allow for the application of forces in the pN regime, which is the relevant regime for many DNA-templated processes.

To study chromatin fibers using single-molecule force spectroscopy, it is necessary to prepare well-defined chromatin constructs. In vitro reconstitution of chromatin has been essential for this purpose, and is typically achieved through salt-gradient dialysis (Luger et al. 1999; Kaczmarczyk et al. 2018). The DNA templates contain multiple Widom 601 nucleosome positioning sequences, separated by linker DNA (Lowary and Widom 1998). Histone octamers preferentially assemble on these 601 sequences, allowing for precise and reproducible nucleosome positioning along the DNA. This controlled assembly is crucial for generating homogeneous chromatin fibers suitable for single-molecule measurements.

Individual chromatin molecules can be tethered between two polystyrene beads (optical tweezers) or a magnetic bead and a surface (magnetic tweezers) **(**Fig. 1C**)**. The fiber can be unfolded by moving the beads with high precision. The applied force is monitored as a function of the extension, resulting in force-distance curves that provide signatures of structural transitions **(**Fig. 1D**)**. For naked DNA, these curves display a smooth profile characteristic of the entropic and enthalpic contributions to its elasticity (Odijk 1995; Marko and Siggia 1995). In contrast, the force-distance curve of chromatin fiber unfolding shows signatures and steps that are associated with unfolding events.

The force-induced unwrapping of nucleosomal DNA has two distinct transitions: first, the nucleosome outer turn unwraps at low forces of ~ 3 pN, followed by unwrapping of the inner turn at forces exceeding ~ 8–9 pN (Mihardja et al. 2006; Chien & van der Heijden, 2014). The low force transition is reversible, as the nucleosome outer turn can be unwrapped and rewrapped repeatedly without notable hysteresis (Brower-Toland et al. 2002; Kruithof et al. 2009). This reflects the dynamic nature of the nucleosome outer turn, showing transient and spontaneous unwrapping of DNA at the entry/exit site as demonstrated with smFRET (Koopmans et al. 2007). This is referred to as site exposure (or ‘breathing’), and is implicated in processes such as transcription factor binding (Donovan et al. 2023) and transcription termination (Hildreth et al. 2020).

In contrast to the nucleosome outer turn, inner turn unwrapping can be observed as discrete steps, each representing unwrapping ~ 75 bp DNA from the periphery of individual nucleosomes (Cui and Bustamante 2000; Bennink et al. 2001a, b). Usually, the first inner turn unwrapping event is observed around ~ 8 pN, with subsequent events in close succession. Counting of these steps can reliably be used to determine the number of assembled nucleosomes on a fiber. Dissociation of histone proteins after inner turn unwrapping impairs complete nucleosome reassembly, although nucleosomes can reassemble if the tension on the fiber is relieved before complete dissociation (Cui and Bustamante 2000; Bennink et al. 2001b); Pope et al. 2005; Spakman et al. 2020).

The assessment of force-distance curves of chromatin unfolding thus offers insights that static images cannot provide. They expose the sequence of unfolding events and transitions, and reveal the forces required to disrupt the structures that define chromatin folding.

## From fibers to higher-order folding

Single-molecule force spectroscopy provides insights beyond the wrapping of nucleosomes. The interactions between nucleosomes can drive a multitude of higher-order folding conformations. The disruption of these interaction occurs at low forces, in a similar range as unwrapping of the nucleosome outer turns. Force spectroscopy enables quantitative analysis of how ionic environment, nucleosome repeat length (NRL), and linker histone stoichiometry shape chromatin topology.

The formation of nucleosomes is driven by electrostatic interactions between the negatively charged backbone of DNA and the positively charged residues at the surface of the histone octamer. However, these interactions only partially neutralize the repulsive electrostatic interactions of nucleosomal DNA, resulting in a high net negative charge for the fully wrapped nucleosome (Gebala et al. 2019). Under low salt conditions, this is combined with additional charge repulsion of the negatively charged linker DNA, resulting in an extended, decompacted conformation of the chromatin fiber (Thoma et al. 1979). When mono- or divalent cations are introduced, this charge repulsion can be shielded, and chromatin can fold into a more compacted fiber (Finch and Klug 1976; Thoma et al. 1979; Widom 1986). Interestingly, even at high concentrations, monovalent cations only induce limited chromatin compaction, resulting in fibers that lack an orderly folded structure (Thoma et al. 1979; Butler and Thomas 1980; Garcia-Ramirez et al. 1992). In contrast, divalent cations, particularly Mg^2+^, exceed the degree of compaction attained with elevated levels of monovalent cations (Widom 1986; Schwarz and Hansen 1994; Korolev et al. 2022). Importantly, this contrasts the moderate compaction of naked DNA at similar concentration ranges, suggesting that Mg^2+^-induced compaction of chromatin arises from mechanisms beyond screening the electrostatic repulsion of the underlying DNA (Korolev et al. 2010, 2022). Indeed, Mg^2+^ promotes intra-fiber nucleosome-nucleosome (‘stacking’) interactions by its increased charge screening due to ion-ion correlation effects (Gelbart et al. 2000) and bridging of histone tails (Korolev et al. 2012), together stimulating interactions between the positively charged residues of the H4 N-terminal tail domain and the acidic patch on the surface of the H2A/H2B dimer (Dorigo et al. 2003; Chen et al. 2017). In both magnetic tweezers and optical tweezers, buffer conditions can easily be exchanged. This allows for systematic testing of the effect of ionic environments on force-distance curves (Chien & van der Heijden, 2014).

**Fig. 2:**
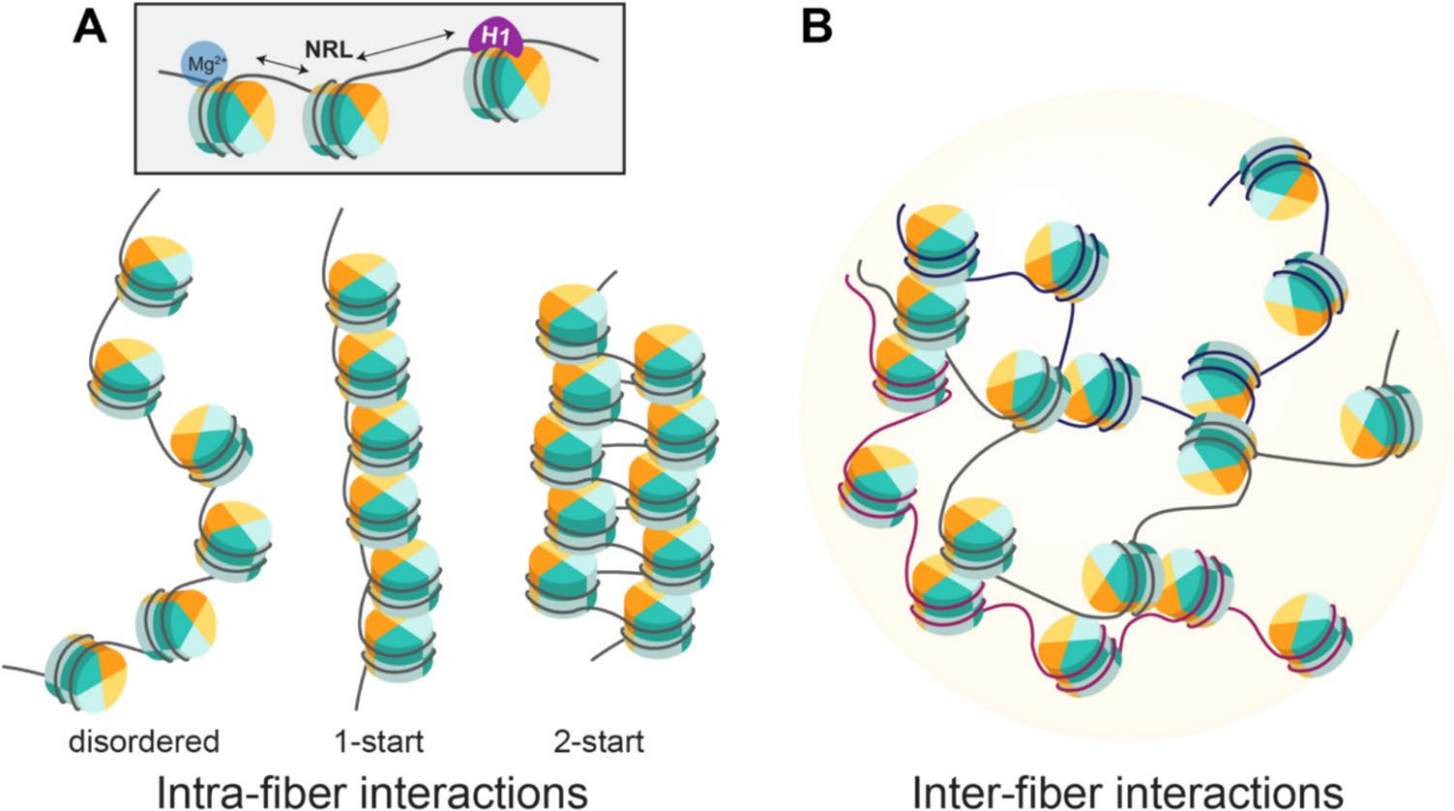
Higher-order chromatin interactions. **A.** Ionic environment, NRL, and linker histones contribute to chromatin topology. Three general topologies are described for chromatin fiber folding. Disordered fibers lack regularity in stacking interactions and folding conformation. 1-start (solenoid) and 2-start (zigzag) fibers form 1 or 2 nucleosome stacks, respectively **B.** Nucleosomal interactions between different fibers are implicated in chromatin condensate formation

Nucleosome repeat length (NRL) reflects the spacing of nucleosomes and is defined as the center-to-center distance between neighboring nucleosomes. DNA has limited flexibility, and the length of linker DNA thus determines whether and how nucleosomes in the same fiber interact. Guided by AFM and (cryo-)EM results, three general architectures are described **(**Fig. 2A**)**: 1-start (solenoid) and 2-start (zigzag) fibers, which form 1 or 2 nucleosome stacks, respectively, and disordered fibers without regular stacking (Schalch et al. 2005; Robinson et al. 2006; Routh et al. 2008; Song et al. 2014). Magnetic tweezers studies systematically comparing NRLs found that NRL197 fibers likely have a 1-start topology, whereas NRL167 fibers are stiffer, consistent with a 2-start arrangement (Kruithof et al. 2009; Kaczmarczyk et al. 2017). Furthermore, comparing the force response of single nucleosomes with chromatin fibers showed that embedding nucleosomes in a chromatin fiber stabilizes them by ~ 10 kbT (Meng et al. 2015). The elastic response of chromatin fibers with crosslinked histone H4 tails showed that fibers with covalently linked nucleosomes feature the same folding characteristics as unmodified chromatin fibers, but with increased resistance to stretching, further confirming that folding topology is dictated by NRL (Kaczmarczyk et al. 2017). Intriguingly, even a single basepair change in linker length can significantly alter or disrupt fiber folding, emphasizing the precision required for stacking interactions in individual chromatin fibers (Brouwer et al. 2021).

Since the histone N-terminal tails mediate nucleosome stacking, they have a profound effect on chromatin folding. Proteolytic removal of histone tails abolishes chromatin folding (Carruthers and Hansen 2000), and selective tail deletions indicate that the H4 tail is uniquely required for maximum compaction (Dorigo et al. 2003). Within the H4 tail, the K16 residue plays an essential role: mutations or charge neutralization of K16 weakens both intra-array folding and inter-array association (Chen et al. 2017). Furthermore, H4K16 acetylation decompacts nucleosome arrays (Shogren-Knaak et al. 2006; Allahverdi et al. 2011), even exceeding the effect of complete H4 tail deletion (Robinson et al. 2008). These results indicate an essential role for histone tails, although the effect of H4 tail deletion on fiber folding depends on NRL (Brouwer et al. 2021).

Linker histone binding cannot compensate for the role of H4 tails (Carruthers and Hansen 2000), but their presence can aid chromatin compaction significantly. Binding of linker histones influences the trajectory of nucleosomal DNA at the entry/exit site (Hamiche et al. 1996; Bednar et al. 1998) and constrains an additional 20 bp of linker DNA (Noll and Kornberg 1977; Allan et al. 1980). Early studies suggested that binding of linker H1 histones to the entry/exit sites of nucleosomes is crucial for the formation of higher-order chromatin conformations (Thoma et al. 1979; Allan et al. 1980; Ausio et al. 1984; Thomas 1999). Magnetic tweezers studies later showed that H1 increases the stability of individual nucleosomes by increasing the free energy required to disrupt the outer turn (Kruithof et al. 2009; Li et al. 2016; Xiao et al. 2018). In addition, single-molecule work also showed that H1 can compact naked DNA (Xiao et al. 2012) and interact with ssDNA (Leicher et al. 2022), pointing to a diverse role in for H1 in chromatin organization.

## Embracing heterogeneity and approximating physiology

Single-molecule studies have provided unprecedented quantitative insights into folding and unfolding of individual chromatin fibers. With the maturing of single-molecule technology and biochemistry, the path is open to assess chromatin fiber folding under conditions that more closely resemble physiology.

Most studies to date have used standardized fibers with equally spaced nucleosomes. These regularly spaced and phased nucleosome arrays are able to form chromatin domains in vitro that resemble domains in vivo (Oberbeckmann et al. 2024), but in living cells, chromatin exists in a heterogeneous environment with mixed ionic conditions and uneven nucleosome spacing (Nishino et al. 2012; Joti et al. 2012; Beel et al. 2021). Recent single-molecule studies took the first steps towards accounting for heterogeneity in fiber composition. Topologically associated domain (TAD)-sized chromatin fibers were reconstituted on either T4 phage – or native source DNA that feature irregularly spaced clutches of nucleosomes (Zinchenko et al. 2018; Korolev et al. 2022). The mechanical properties of such fibers are comparable with that of shorter 601 arrays, and also show large rupture events that correspond to the breaking of interactions between distant clutches of nucleosomes (Korolev et al. 2022). Physiological Mg^2+^ concentrations compact such fibers in vitro, with maximum condensation of chromatin fibers of vastly different sizes and compaction geometries, such as T4 DNA-based arrays (~ 166 kb), λ-phage DNA-based arrays (~ 48.5 kb) and 12-mer nucleosome arrays occurring in a very similar range of Mg^2+^ concentrations (from 2 to 4 mM) (Allahverdi et al. 2015; Zinchenko et al. 2018; Korolev et al. 2022). This suggests that folding mechanisms for chromatin fibers of various sizes and nucleosome arrangements are inherent to the properties of chromatin itself. Another study used magnetic tweezers to characterize telomeric chromatin fibers, revealing these fibers fold into a columnar structure (Soman et al. 2022). These examples are important steps towards applying quantitative force spectroscopy to native chromatin structure.

The role of histone tails in chromatin folding is clear, but less is known about the effect of individual histone modifications. Tails are the prime site of post translational modifications including methylation, acetylation, ubiquitination, sumoylation, and many more (Millán-Zambrano et al. 2022). The effect of a number of these have been explored and quantified with single-molecule studies (Brower-Toland et al. 2002, 2005; Allahverdi et al. 2011; Simon et al. 2011; Lin et al. 2024), but as the list of modification grows, including modifications on the globular domains of histones, their quantified effects are lacking. Similarly, histone variants beyond the canonical octamer could provide insight into specific biological roles. For example, centromere-specific nucleosome CENP-A was shown to have similar stability to canonical octamers (Kim et al. 2016). Access to in vitro modified histone octamers will likely aid this process.

Chromatin in the nucleus is further organized into higher-order structures on various length scales including loops, domains and compartments (Gibcus and Dekker 2013). Organization on these scales is driven by longer scale and inter-fiber interactions, and often involve multivalent interactions. In recent years, the formation of biomolecular condensates has been proposed as such a multivalent organisational mechanism. Indeed, chromatin can form biomolecular condensates in vitro and in vivo, either by itself or in combination with binding partners (Larson et al. 2017; Strom et al. 2017; Gibson et al. 2019; Sanulli et al. 2019) **(**Fig. 2B**)**. This process has been studied primarily with imaging-based methods on in vitro systems using reconstituted chromatin fibers, often built upon foundational insights derived from single-molecule force spectroscopy. For example, the formation of chromatin condensates strongly depends on linker length, and thereby the ability of the nucleosomes within the same fiber to interact: chromatin fibers that favor stacking interactions have a weaker propensity to form condensates (Gibson et al. 2019; Chen et al. 2025). This implies that interactions between different fibers are the driving force for condensate formation. The interaction between the histone H4 tail and the acidic patch of the octamer induces nucleosomal interactions both within individual fibers (Dorigo et al. 2003; Chen et al. 2017) and between different fibers (Kan et al. 2009). In addition, decompaction of chromatin by H4 tail acetylation and acidic patch neutralization has also been implicated in inter-fiber interactions (Shogren-Knaak et al. 2006; Berezhnoy et al. 2016), and histone acetylation antagonizes chromatin condensate formation (Gibson et al. 2019). Although this implies that intra- and inter-fiber H4 tail-acidic patch interactions are locally mutually exclusive, on the nuclear scale, it is likely that both interaction types coexist, with ionic conditions contributing to their regulation. In an analytical ultracentrifugation study where the composition of the mixture of cations in the nucleus was mimicked, it was found that in the presence of Mg^2+^, Na^+^ promotes condensation of nucleosome arrays into organized structures, but K^+^ abrogates folding of individual fibers (Allahverdi et al. 2011, 2015). This contrasts self-association of nucleosome arrays in bulk under mixed salt conditions, which is synergistically promoted by Mg^2+^ and Na^+^ or K^+^ (Allahverdi et al. 2015). This highlights that changes in the nuclear environment possibly favour one type of interaction over the other, thereby attaining an additional layer of organization that is dependent on cell state.

To date, studies on chromatin condensate formation and properties have relied predominantly on microscopy-based approaches. However, optical tweezers offer a powerful alternative for probing fusion dynamics of condensates and further elucidating their material properties (Vizjak et al. 2024; Kamp et al. 2025). This approach enables precise mechanical manipulation and quantitative measurements that are difficult to achieve using imaging techniques alone.

## Conclusion and perspective

Single-molecule studies have provided detailed insights into factors that influence chromatin fiber organization and their unfolding dynamics, contributing to our understanding of larger scale chromatin folding inside living cells. Although force spectroscopy has provided significant insights into the mechanical behavior of chromatin, several technical and conceptual challenges remain that limit broader applicability and impact. For example, sample preparation remains non-trivial and often requires highly optimized and carefully controlled protocols that are difficult to standardize and scale, resulting in limited experimental throughput. Future progress depends on the application of robust and reproducible sample preparation and optimized experimental procedures. The implementation of multiplexed approaches, advances in bead tracking algorithms and microfluidic platforms have already significantly increased throughput (Enger et al. 2004; Gross et al. 2010; De Vlaminck et al. 2011). In addition, collaboration with computational experts should focus on further refining existing models of folding topology and potentially developing new ones. Collectively, these advances are essential for interpreting force spectroscopy data and will contribute to more efficient and streamlined data analysis.

Conventional force spectroscopy provides primarily mechanical readouts and offers limited direct information about the underlying structural transitions. An important step forward is the integration of correlative optical tweezers with fluorescence microscopy (Chua and Liu 2024). This enables real-time visualization of binding events and structural transitions in conjunction with force measurements. This approach is highly suitable to characterize chromatin interactors and study the mechanisms of chromatin remodelers (Carcamo et al. 2022; Spakman et al. 2022; Buzón et al. 2023). Capturing the intrinsic heterogeneity of chromatin observed in cells also remains a major challenge. While reconstituted systems are powerful to systematically study specific components that influence the organization of single fibers or chromatin condensates, they only partially reflect the composition and structural diversity of chromatin in its native state. Ultimately, closer integration with complementary biochemical, structural, imaging and sequence-based approaches will be critical to converge in vitro findings with observations of chromatin organization in the nucleus. Using native chromatin fibers and experimental conditions that better approximate physiological environments contribute to a more unified and mechanistic understanding of chromatin organization. Excitingly, these first steps towards bringing the in vitro results and in vivo chromatin structure closer together have been taken. Future work will further reconcile the different length scales and help us understand how heterogeneity links to function.

## Data Availability

No datasets were generated or analysed during the current study.
